# Antimicrobial Efficiency of ‘Green’ Silver Nanoparticles Against Plant and Human Pathogens for Environmental Sanitation

**DOI:** 10.3390/ma18214952

**Published:** 2025-10-30

**Authors:** Svitlana Dybkova, Konrad Terpilowski, Olena Goncharuk, Mykhaylo Dybkov, Liudmyla Rieznichenko, Olha Liutko, Kateryna Vitrak, Tamara Gruzina, Katarzyna Szewczuk-Karpisz

**Affiliations:** 1 F.D. Ovcharenko Institute of Biocolloidal Chemistry, National Academy of Science of Ukraine, 42 Vernadskogo Blvd., 03142 Kyiv, Ukraine; sdybkova@gmail.com (S.D.); lrieznichenko@gmail.com (L.R.); gruzinatamara@gmail.com (T.G.); 2 Department of Interfacial Phenomena, Maria Curie-Sklodowska University, M. Curie-Sklodowska Sq. 3, 20031 Lublin, Poland; 3 Institute of Agrophysics, Polish Academy of Sciences, Doświadczalna 4, 20290 Lublin, Poland; k.szewczuk-karpisz@ipan.lublin.pl; 4 The Institute of Molecular Biology and Genetics, National Academy of Science of Ukraine, 03143 Kyiv, Ukraine; mdybkov@edu.imbg.org.ua; 5 SI “The Institute of Traumatology and Orthopedics”, National Academy of Medical Sciences of Ukraine, 01601 Kyiv, Ukraine; o.liutko@gmail.com (O.L.); k.vitrak1122@gmail.com (K.V.)

**Keywords:** silver nanoparticles, green synthesis, antimicrobial activity, soil sanitation, sanitary-indicator bacteria, phytopathogenic bacteria, *Pseudomonas syringae*, *Zea mays*, nanobiotechnology

## Abstract

Silver nanoparticles (AgNPs) synthesized by ‘green’ methods using plant extracts have emerged as promising antimicrobial agents for combating soilborne pathogens. In this study, the antimicrobial activity of four AgNP formulations prepared using various reducing agents (AgNP#1, AgNP#2, AgNP#3, AgNP#4) against sanitary-indicator bacteria (*Escherichia coli* ATCC 25922, *Enterococcus faecalis* ATCC 29213, *Staphylococcus aureus* ATCC 25923, *Pseudomonas aeruginosa* ATCC 27853) and phytopathogenic *Pseudomonas syringae* strains isolated from *Zea mays* plants was estimated. The results demonstrated that AgNP#3 and AgNP#4 exhibited the greatest antibacterial efficacy, with minimal inhibitory concentrations (MIC). The soil incubation studies confirmed that AgNPs reduced the population of *P. syringae* without significant effects on beneficial soil microbiota. AgNP#1 and AgNP#2 exhibited a stimulatory effect on the *Zea mays* seed germination, bringing out their potential for agricultural applications. Thus, the developed biogenic AgNPs could serve as efficient antimicrobial agents for sustainable soil sanitation while minimizing environmental risks.

## 1. Introduction

Soil serves as a critical reservoir for various microorganisms, facilitating their transmission to humans and animals. The application of organic farming practices, particularly the use of livestock farm waste as fertilizer, increases the risk of pathogen proliferation in arable soils [1]. Soil contamination with animal waste contributes to the dissemination of antibiotic-resistant bacteria and zoonotic diseases [2]. Even well-composted manure can harbor *Escherichia coli* O157:H7 and *Salmonella* spp., which remain viable for months in soil [3]. Pathogens such as *Clostridium* spp., *Salmonella* spp., *E. coli*, and *Listeria monocytogenes* persist in soil for extended periods [4]. Thus, sustainable soil management practices, such as pre-treatment of organic fertilizers, are crucial for mitigating microbial risks [5]. Developing effective soil sanitation methods using antiseptics that are non-toxic to eukaryotic organisms is currently a pressing need.

Modern sanitary and epidemiological practices are focused on protecting soil from contamination by pathogenic microorganisms. Chemical and biological antiseptics are used to sanitize soil. Traditional disinfectants, such as chlorine compounds and hydrogen peroxide, can be effective, but they can also disrupt soil microbial communities. Silver nanoparticles (AgNPs) and plant-based antimicrobials have emerged as alternative nanopesticides due to their selective toxicity to pathogens while preserving beneficial microbes [6,7,8]. AgNPs exhibit strong antimicrobial activity against a broad spectrum of bacteria, fungi, and viruses [9,10,11]. Their mechanism of action is to break down microbial cell membranes, produce reactive oxygen species (ROS), and disrupt DNA replication [12]. Some researchers proved AgNPs’ effectiveness against the gram-negative and gram-positive phytopathogenic bacteria, including *E. amylovora*, *C. michiganensis*, *R. solanacearum*, *X. campestris* pv. *campestris*, and *D. solani*, which are responsible for significant crop losses [13] as well as *P. syringae* [14], *Pythium* sp., *Colletotrichum* sp., *X. compestris*, *B. sorokiniana*, *M. grisea*, *F. culmorum*, etc. [15]. Among the mechanisms that can generally influence the interactions of AgNPs with GPB and GNB, it should be noted that the differences in the structure of their cell membrane should be pointed out. Gram-negative bacteria have an outer membrane with lipopolysaccharides, which acts as a barrier limiting penetration of AgNPs [16]. In turn, the GPBs have a thicker peptidoglycan layer, more permeable to nanoparticles. Lamsal et al. [17] confirmed that *S. aureus* is more sensitive to AgNPs than *E. coli*. Similar results were obtained by Morones et al. [12], who proved that *P. aeruginosa* had greater resistance to AgNPs due to its outer membrane. The lack of an outer membrane in the GPB structure, as well as the presence of a thick peptidoglycan layer, can allow AgNPs to interact more readily with the cell wall, making these bacteria more susceptible to the AgNP-induced damage. The mechanisms of AgNPs’ action depend on the structure of the bacterial cell and AgNPs’ properties, namely, size, concentration, crystal structure, and stabilizing agents. AgNPs can adsorb onto the surface of the bacterial cell, disrupting the integrity of the cell wall and membrane, leading to a loss of ionic balance and membrane permeability. This can cause cell lysis [18]. In addition, silver ions (Ag^+^) released from nanoparticles can bind to the thiol groups of proteins, inhibiting their functions [19]. Once inside the bacterial cell, AgNPs and Ag^+^ ions can interact with DNA and ribosomes, inhibiting DNA replication and protein synthesis as well as affecting metabolism. Thus hindering bacterial growth and leading to cell death. AgNPs can also induce the production of ROS, such as superoxide radicals and hydrogen peroxide, leading to oxidative stress [19]. This damages cellular components, including lipids, proteins, and DNA, ultimately resulting in bacterial cell death. Finally, AgNPs can interfere with bacterial energy production by disrupting the electron transport chain and ATP synthesis. This interaction leads to energy depletion and impairs bacterial viability.

In addition to controlling soil-borne pathogens, AgNPs contribute to plant health and growth improvement. These species can improve seed germination and plant resistance to bacterial infections [17]. Thus, their incorporation into hybrid hydrogels should also be beneficial, i.e., reducing microbial contamination without significant improvement of soil microorganisms [20]. Biologically synthesized AgNPs sometimes have a more pronounced antimicrobial effect compared to AgNPs synthesized using chemical reducing agents. After their application, the number of soil bacterial populations was significantly reduced, and minimal impact on the antibiotic resistance of soil microbiota was noted [21,22]. The controlled release of AgNPs through nanocomposites further enhances their efficacy and minimizes environmental risks [23]. However, concerns regarding their long-term impact on soil microbial diversity and ecosystem balance warrant further investigations [24]. For example, AgNPs can pose a threat to soil bacteria, damaging their cell wall. Moreover, the delayed effect of AgNPs on living organisms, including humans, animals, and plants, is particularly important [25,26,27,28].

In the previous paper, the influence of ‘green’ synthesis conditions and structural features of the obtained AgNPs on their biosafety was tested [29]. The prospects for the use of ‘green’ AgNPs to solve environmental problems due to the absence of geno- and cytotoxicity were pointed out. The AgNPs’ effectiveness against soil pathogens remained unexplored. As a result, it is still unknown whether AgNPs can be incorporated into hydrogels intended for soil applications. To fill this literature gap, the effect on pathogens in such agroecosystems as *E. coli*, *E. faecalis*, *S. aureus*, *P. aeruginosa,* and *P. syringae*, posing a serious threat to crop production and human health, as well as on the specific plant (*Zea mays*) pathogen, was investigated in the current study. These microorganisms can cause plant diseases, reduce yield and product quality, and be a source of infections for humans.

## 2. Materials and Methods

### 2.1. Silver Nanoparticles (AgNPs) Formulations

A series of AgNPs was prepared using environmentally friendly (“green”) reduction methods employing plant-derived extracts as reducing and stabilizing agents [29]. AgNP#1 was synthesized by reducing silver nitrate with a water-alcohol extract of *Eucalyptus viminalis* Labill leaves in the presence of potassium carbonate. An aqueous solution of AgNO_3_ was mixed with the K_2_CO_3_ solution, followed by the addition of the alcohol–water extract. The mixture (pH ≈ 5.2) was subjected to autoclaving at 121 °C and 1.04 atm for 15 min to complete the reduction. AgNP#2 was obtained by reacting silver nitrate with the aqueous *Eucalyptus viminalis* Labill leaf extract. The AgNO_3_ solution and plant extract (pH ≈ 4.4) were combined and autoclaved under the same conditions. AgNP#3 was formed by reducing silver nitrate with the tannin solution in the presence of potassium carbonate (pH ≈ 5.9), using the identical autoclaving conditions. AgNP#4 was prepared at room temperature by adding the aqueous aloe (*Aloe arborescens* Mill) extract to a silver-ammonia complex solution (pH ≈ 8.4), with the final volume adjusted using distilled water.

These procedures utilize renewable plant materials, avoid toxic chemical reducing agents, and follow mild reaction conditions, consistent with the principles of green nanoparticle synthesis.

The structural characterization of AgNPs was performed using transmission electron microscopy (TEM, Tecnai G2 T20 X-TWIN, FEI Company, Hillsboro, OR, USA) operating at an accelerating voltage of 200 kV with an electron beam source. 5 μL of the AgNP formulations was carefully deposited onto carbon-coated copper TEM grids and left to air-dry under ambient conditions.

### 2.2. Antimicrobial Activity of AgNPs Against Indicator Bacteria

The antimicrobial activity of AgNPs against sanitary-indicator bacteria was studied using the disk diffusion method [12,13,30,31] with the bacterial strains obtained from the Laboratory of Microbiology and Chemotherapy of the State Institution “Institute of Traumatology and Orthopedics of the National Academy of Medical Sciences of Ukraine” (Kyiv, Ukraine): *Escherichia coli* ATCC 25922; *Enterococcus faecalis* ATCC 29213; *Staphylococcus aureus* ATCC 25923; *Pseudomonas aeruginosa* ATCC 27853.

Bacterial cultures were grown in the liquid nutrient medium Luria Bertani broth (LB, Condalab, Torrejón de Ardoz, Spain) for 18–20 h at 37 °C. A standard inoculum corresponding to the 0.5 McFarland standard was used. A 100 µL bacterial suspension was spread over the surface of Mueller-Hinton agar plates using a sterile spreader. Sterile 6 mm diameter paper discs were placed on the agar surface with sterile tweezers, and 20 µL of the tested silver nanoparticle formulations were applied to each disc. Then the Petri dishes were incubated at 37 °C for 24 h. After incubation, the diameters of the inhibition zones were measured to the nearest millimeter, considering the area of complete bacterial growth suppression.

The minimum inhibitory concentration (MIC) and the minimum bactericidal concentration (MBC) of the tested AgNPs against the sanitary-indicator pathogenic and opportunistic bacterial strains were determined using a resazurin assay [32]. A nutrient medium (LB), a bacterial inoculum of 10^6^ CFU/mL, and various concentrations of the nanoparticles under study were added to sterile test tubes. The total volume of the mixtures was 0.3 mL. The negative control was the uninoculated medium, and the positive control was the medium inoculated with bacteria. Thirty microlitres of resazurin solution were added to each test tube and incubated at 37 °C for 24 h. No colour changes were observed. A blue-violet colour indicated the absence of bacterial growth, whereas a pink or colourless appearance indicated bacterial growth. The MIC value was taken as the lowest concentration of antibacterial agents that inhibited bacterial growth (the colour remained blue). Samples from tubes where the medium remained blue were cultured on a solid nutrient medium, and the presence or absence of bacterial growth was recorded. The absence of growth indicated the established MIC.

### 2.3. Antimicrobial Activity of AgNPs Against Phytopathogenic Bacteria

The antimicrobial activity of AgNPs against the phytopathogenic bacterium *Pseudomonas syringae* was studied using two strains of this species isolated from infected *Zea mays* plants in Ukraine (PE “KARATUL SEEDS” Ukraine, Kyiv region, Velyka Karatul).

To isolate *P. syringae* phytopathogens, leaves and *Zea mays* ears showing signs of infection were collected. Infected tissue fragments (1 g) were ground and resuspended in 2 mL of 0.56% NaCl solution, pH 6.5. A 0.1 mL suspension was spread on Luria Bertani agar (LA, Condalab, Spain) and incubated for 24–48 h at 21–24 °C. The colonies growing on the agar surface were characterized morphologically. Further identification of bacterial isolates was performed based on the biochemical properties and the presence of a characteristic phytopathogenic gene. Strains identified as *P. syringae* were stored in a laboratory collection for subsequent antimicrobial testing of AgNPs.

Identification of *P. syringae* was made as follows. DNA was extracted from the obtained isolates by prolonged heating (boiling) of an aqueous bacterial suspension at 95 °C. *P. syringae* was identified by detecting a characteristic phytopathogenic Psy gene using a specific pair of oligonucleotide primers [33]: (i) Psy_F ATGATCGGAGCGGACAAG and (ii) Psy_R GCTCTTGAGGCAAGCACT

PCR was performed in a 25 µL reaction mixture containing 0.5 µg DNA and 10 pmol of each primer. The amplification conditions were: 1 cycle at 93.5 °C for 8 min, followed by 40 cycles of 93.5 °C for 35 s, 55 °C for 35 s, and 72 °C for 40 s. PCR products were separated in a 1.8% agarose gel and visualized using ethidium bromide staining. The fragment size corresponding to the Psy gene was 144 bp.

The antimicrobial activity of AgNPs against *P. syringae* was assessed using the disk diffusion method, similar to the testing of the sanitary-indicator bacterial strains (*E. coli*, *E. faecalis*, *S. aureus*, *P. aeruginosa*). Cultures of *P. syringae* strains were grown in Luria Broth for 18–20 h at 21 °C. MBC values for AgNP against *P. syringae* were determined using the serial dilution method in Luria Broth, followed by plating on Luria Agar. The samples from tubes exhibiting no visible bacterial growth were inoculated onto the LA plates and incubated for 24 h at 21 °C. MBC was determined as the lowest AgNP concentration at which no bacterial growth was observed on LA.

Statistical analysis of antimicrobial activity was performed by calculating mean values and standard deviations, following standard protocols for biological studies. The significance level for all statistical tests was set at *p* < 0.05.

### 2.4. Soil Analysis and Antimicrobial Testing in Soil

Ukrainian soil classified as sandy silt loam was used in the study. The soil was characterized as neutral in terms of pH (water and hydrolytic), with optimal salinity, low organic matter content, moderate cation exchange capacity (CEC), and very low exchangeable potassium levels.

The antimicrobial dynamics of AgNPs against *P. syringae* in soil were studied by introducing 1 × 10^7^ CFU/g soil of both phytopathogen strains and AgNPs at their respective MBCs into sterile soil (autoclaved at 121 °C for 30 min). The inoculated soil was incubated at 21 °C. Microbiological control of *P. syringae* populations was conducted by plating aliquots of the soil onto LA medium after 4, 8, and 12 days of incubation.

The antimicrobial activity of AgNP against soil microbiota was assessed by introducing AgNPs at their MBCs into the non-sterile soil. Soil was incubated at 21 °C, and microbial populations were monitored by counting colony-forming units (CFU) on LA after 4, 8, and 12 days of incubation.

The method for determining CFU (colony-forming units) was based on microbiological culture of the sample on a solid nutrient medium, followed by incubation and counting of the formed colonies. For this purpose, the soil extract sample was applied in various dilutions (10^−4^–10^−8^) to into a Petri dish with a nutrient medium (LA), incubated for 24–48 h in a thermostat at a temperature of +21–24 °C, and then the formed colonies were counted. The resulting number was used to calculate the CFU per unit of soil mass.

### 2.5. Antimicrobial Activity in P. syringae: Contaminated Soil with Zea mays Seed Germination

The antimicrobial effects of AgNPs in the *P. syringae*-contaminated soil were studied during *Zea mays* seed germination. 100 g of non-sterile soil was inoculated with *P. syringae* at 1 × 10^7^ CFU/g, treated with AgNPs at their MBCs, and sown with five *Zea mays* seeds. Germination was conducted at 15–17 °C, which is considered ‘friendly germination’ under the agronomic standards and lasts up to 15 days. On day 16, the presence of seedlings was recorded, and stem length as well as morphological features were measured. The control group included untreated soil with the *Zea mays* seedlings.

All experiments were conducted in duplicate with two technical replicates.

## 3. Results

### 3.1. Antibacterial Activity of Silver Nanoparticles Against Sanitary-Indicator Bacteria

**Figure 1:**
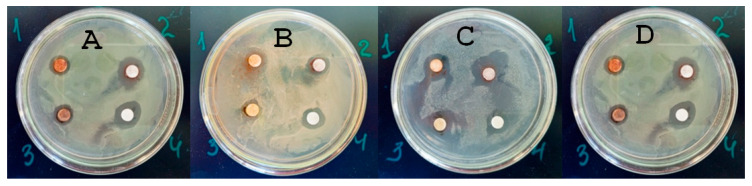
Antimicrobial activity of AgNPs (1—AgNP#1, 2—AgNP#2, 3—AgNP#3, 4—AgNP#4) assessed based on the disk diffusion method against the sanitary-indicator microorganisms: (**A**)—*E. coli* ATCC 25922, (**B**)—*E. faecalis* ATCC 29213, (**C**)—*S. aureus* ATCC 25923, (**D**)—*P. aeruginosa* ATCC 27853.

The study of antimicrobial activity by the disk diffusion method (Table 1) as well as the resazurin test showed a significant antimicrobial activity of the developed AgNPs against the selected indicator microorganisms: *E. coli* ATCC 25922, *E. faecalis* ATCC 29213, *S. aureus* ATCC 25923, and *P. aeruginosa* ATCC 27853. Figure 1 presents the data of the disk diffusion test.

The obtained data indicated a large antimicrobial activity of AgNP#1, AgNP#3, and AgNP#4 against all tested sanitary-indicator microorganisms. In contrast, AgNP#2 did not demonstrate antimicrobial activity against *E. faecalis* ATCC 29213, making it not appropriate for soil decontamination from these pathogens.

The sensitivity of the studied bacterial strains to the tested nanosilver formulations was similar: *E. faecalis* and *S. aureus* were most sensitive to AgNP#1, while the sensitivity of *P. aeruginosa* and *E. coli* to AgNP#1, AgNP#3, and AgNP#4 was the same. Gram-negative bacteria (GNB) have an outer membrane containing lipopolysaccharides, which makes it difficult for antibacterial agents, such as AgNPs, to penetrate. On the other hand, Gram-positive bacteria (GPB) have a thick peptidoglycan layer, which can also affect their susceptibility to antimicrobial agents. As follows from the data in Table 1 and Table 2, AgNP#1, AgNP#3, and AgNP#4 demonstrated antimicrobial activity against both GNB and GPB. *P. aeruginosa* (GNB) was the most sensitive to all AgNPs, which have the largest inhibition of growth zones (in the range of 6–12 mm). In turn, *E. coli* was the least sensitive, exhibiting the smallest inhibition zones (10–11 mm).

Minimum inhibitory concentration (MIC) and minimum bactericidal concentration (MBC) of AgNPs were determined using the resazurin assay. The obtained results are summarized in Table 2. The MIC values of the AgNPs formulations synthesized using alcohol and aqueous extracts of eucalyptus leaves, as well as tannin, ranged from 7.5 to 23 µg/mL (based on silver content), while the MBC parameter ranged from 23 to 30 µg/mL for all tested strains. The MIC of the aloe leaf extract-based formulation was 1.5 µg/mL, and its MBC ranged from 1.5 to 3.5 µg/mL. The results of studies of antimicrobial activity, i.e., the disc diffusion assay and the MIC and MBC tests, coincided in the case of AgNP#1, AgNP#3, and AgNP#4. For AgNP#2, despite the fact that the results of the disc diffusion test showed the absence of growth inhibition for *E. coli* ATCC 25922 and *E. faecalis* ATCC 29213, the MIC method indicated that the growth inhibition occurred at 15 µg/mL for *E. coli* ATCC 25922 and 23 µg/mL for *E. faecalis* ATCC 29213, whereas the MBC values were equal to 23 and 30 µg/mL, respectively.

This can be dictated by the imperfection of the disk diffusion test for determining the antimicrobial activity of metal nanoparticles. Additional comprehensive studies should be carried out, as presented below. The MIC parameter of AgNP#1, AgNP#3, and AgNP#4 was the lowest for *S. aureus* ATCC 25923, indicating the greatest sensitivity of these bacteria to AgNPs. The gram-negative bacteria, *P. aeruginosa* and *E. faecalis*, required almost the same concentrations of AgNPs to achieve a bactericidal effect. This indicated the universality of the AgNPs’ antimicrobial activity.

**Figure 2:**
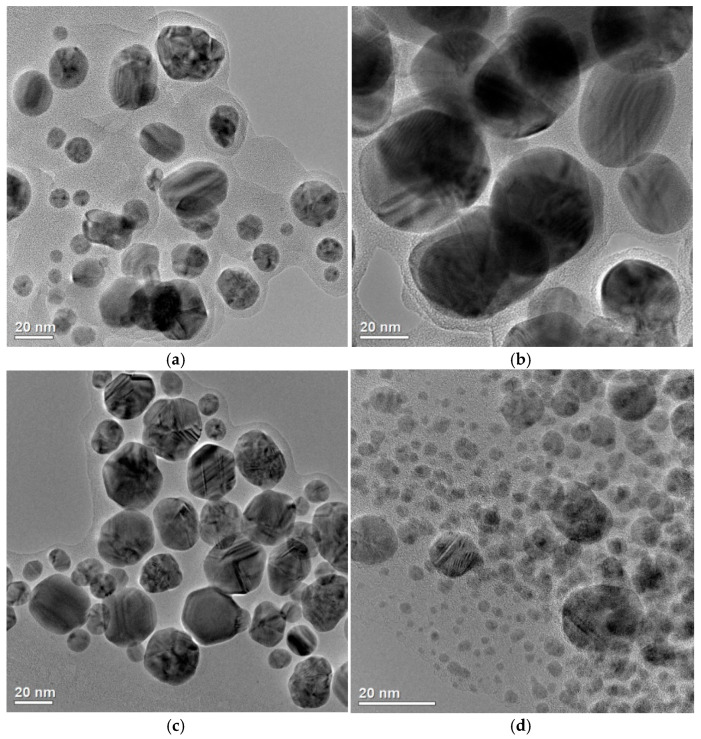
TEM images of the studied AgNPs formulations: (**a**)—AgNP#1, (**b**)—AgNP#2, (**c**)—AgNP#3, and (**d**)—AgNP#4.

According to the general trends reported in the literature [34,35,36], the antimicrobial properties of AgNPs depend largely on their size and morphology. Reducing the AgNPs’ size contributes to an increased specific surface area, which enhances the NPs’ interactions with microorganisms and consequently, their antimicrobial activity. The developed AgNPs [29] were synthesized using ‘green’ methods and differed in size and aggregation degree (Figure 2).

AgNP#1 and AgNP#3 had a relatively narrow size distribution, with the particle diameters ranging from 10 to 35 nm, while AgNP#2 exhibited a broader size range—from 15 to 60 nm. AgNP#4 was characterized by the smallest individual nanoparticles (below 10 nm), but showed a higher degree of aggregation. The synthesized AgNPs exhibited a predominantly face-centered cubic crystalline structure, which is characteristic of metallic silver; namely, AgNP#1 and AgNP#3 displayed greater crystallinity with sharper and more intense peaks, suggesting a well-ordered atomic arrangement and lower levels of internal defects. AgNP#2 exhibited broader diffraction peaks, indicating a smaller crystallite size and potentially higher structural disorder. AgNP#4 showed lower crystallinity. As follows from Figure 2 and our previous studies [29], the AgNP#2 formulation has the largest particle size with a diameter exceeding 20 nm, which may be the reason for the lack of its antimicrobial activity against *E. coli* ATCC 25922 and *E. faecalis* ATCC 29213 (Table 1). At the same time, the lowest MIC and MBC values were observed for AgNP#4, which is characterized by the smallest particle size. AgNP#4 also showed the lowest (4 µg/mL) non-toxic concentration, i.e., this formulation had the largest cytotoxicity. Therefore, when choosing the optimal drug, one should be guided not only by its antimicrobial activity indicators, but also by geno- and cytotoxicity.

Regarding cytotoxicity and genotoxicity, all synthesized AgNPs were biosafe, with no significant genotoxic effects [29]. However, cytotoxicity varied among the samples. AgNP#3 had the highest non-toxic concentration (30 µg/mL), while AgNP#4 showed the lowest one (4 µg/mL), indicating a larger cytotoxic effect. AgNP#1 and AgNP#2 were characterized by intermediate cytotoxic thresholds (15 µg/mL and 10 µg/mL, respectively). Thus, the synthesis method significantly influenced not only the size and morphology of nanoparticles, but also the biological safety of AgNPs. AgNP#3 exhibited the most favourable balance of stability, low aggregation, and minimal cytotoxicity. The bactericidal and bacteriostatic activities of this formulation proved to be optimal.

### 3.2. Impact of AgNPs on the Soil Microbiota

Extensive studies are currently being carried out on the penetration of metallic nanoparticles (M-NPs) into air, water, and soil ecosystems. It is known that in soil M-NPs, in particular AgNPs, exhibit different degrees of dissolution, agglomeration, and aggregation depending on their properties (size, shape, surface charge) and physicochemical characteristics of the soil (pH, ionic strength, amount of organic matter, clay content, etc.). These factors determine their mobility and bioavailability to soil organisms, and consequently, their potential toxicity to soil microbiota [37,38,39]. It was proved that at the concentrations 1–2 ppm, AgNPs did not affect aerobic microbial denitrification, indicating a lack of negative impact on soil microflora [40]. However, according to other researchers, AgNPs can decrease the abundance of *Sphingomonas* and *Lysobacter*, while increasing the populations of *Flavobacterium* and *Niastella* in soil layers with a larger metal content [41,42,43]. Taking it into account, it is necessary to assess the possible negative impact of the tested AgNPs formulations on the soil microbiota.

Thus, the next phase of this study focused on the estimation of antimicrobial effects of AgNPs at MBC against sanitary-indicator microorganisms on the naturally occurring soil microbiota. The impact of the tested AgNPs formulations was assessed in sandy silt loam soil, characterized by its pH, salinity, organic matter content, exchangeable calcium and magnesium, cation exchange capacity (CEC), and exchangeable potassium levels.

The effect of the AgNPs formulations on bacteria in the soil was studied, which, according to the indicators of water pH and hydrolytic pH, salinity, organic matter level, exchangeable calcium and exchangeable magnesium, cation exchange capacity, and exchangeable potassium, was identified as the sandy silt loam. The dynamics of total bacterial abundance in the selected soil after exposure to AgNPs are shown in Table 3.

It was observed that the application of all tested AgNPs formulations did not cause a decrease in the total number of soil microorganisms, even though these NPs were applied at the bactericidal concentrations for *E. coli*, *E. faecalis*, *S. aureus*, and *P. aeruginosa* (the concentrations of AgNP#1, AgNP#2, AgNP#3 were 30 µg/g soil, and one of AgNP#4 was 3.5 µg/g soil). A positive effect of the tested AgNPs on soil bacteria was found. AgNP#3 stimulated an increase in the total microbial population; that is, the total bacterial number in the soil increased 1.74-fold in 15 days after the AgNP#3 application compared to the control. A similar stimulating effect was noticed for AgNP#4. Thus, it can be concluded that none of the studied AgNPs caused inhibition of the vital activity of soil bacteria, even in the bactericidal concentrations for sanitary indicator bacteria.

### 3.3. Antibacterial Activity of AgNPs Against Pseudomonas syringae

**Figure 3:**
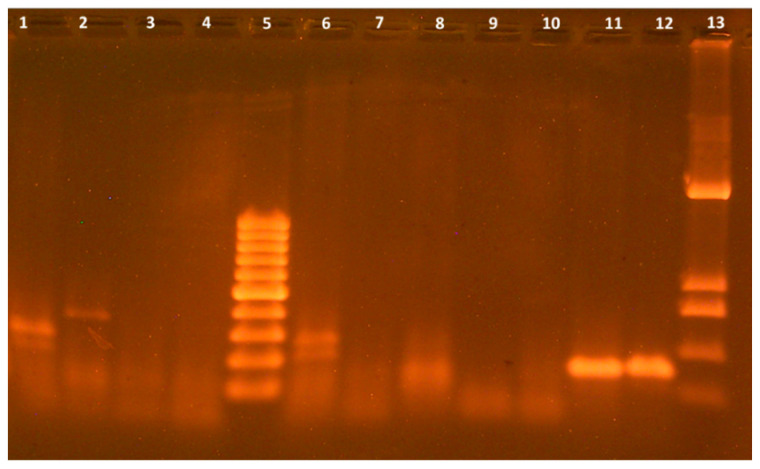
Electrophoregram of PCR products of Psy gene detection: 1—isolate #1, 2—isolate #2, 3—isolate #3, 4—isolate #4, 5—molecular mass marker Generunner 100 bp, 6—isolate #5, 7—isolate #6, 8—isolate #7, 9—isolate #8, 10—isolate #9, 11—isolate #10, 12—isolate #11, 13—molecular mass marker pUC19/Hinf1 (1419, 517, 396, 214, 75, 65 bp).

At the next stage of the study, the antimicrobial properties of AgNPs against phytopathogenic bacteria were determined. 11 isolates of potentially phytopathogenic bacteria were isolated from infected *Zea mays* leaves and ears. To determine isolates that would have a known phytopathogenic gene Psy of bacteria *P. syringae*, the PCR analysis was performed using specific oligonucleotide primers for the specific determination of the Psy gene. As shown in Figure 3, in the two studied isolates, N10 and N11, there were DNA fragments, the size of which corresponded to the Psy gene being 144 bp. The bacteria of isolates N10 and N11 were registered as the laboratory collection of the F.D. Ovcharenko Institute of Biocolloidal Chemistry of the NAS of Ukraine, strain *P. syringae* N1 and strain *P. syringae* N2, respectively.

The bacteria of strain N1 were isolated from *Zea mays* cobs with signs of bacterial disease (blackening of grains, soft, sometimes rotten grains). On the surface of LAgar, after 24 h of cultivation, bacteria of this strain grew in the form of large white colonies with a diameter of up to 6 mm with a smooth surface, whereas in LBroth after 18–24 h—evenly without films and conglomerates. Microorganisms of strain N2 were isolated from corn leaves with signs of bacterial disease (dry spotted fragments).

On the surface of LAgar after 24 h of cultivation, bacteria of this strain grew in the form of small yellowish-white colonies with a diameter of 3 mm, with a smooth surface, whilst in LBroth after 18–24 h, with the formation of films. Both strains were Gram-negative, motile rods, non-spore-forming, oxidase-negative, unable to reduce nitrates, and did not produce hydrogen sulfide or indole. They did not utilize glucose or salicin anaerobically, consistent with *P. syringae* characteristics. The strains showed weak production of fluorescent pigments and utilized glucose and galactose as carbon sources.

**Figure 4:**
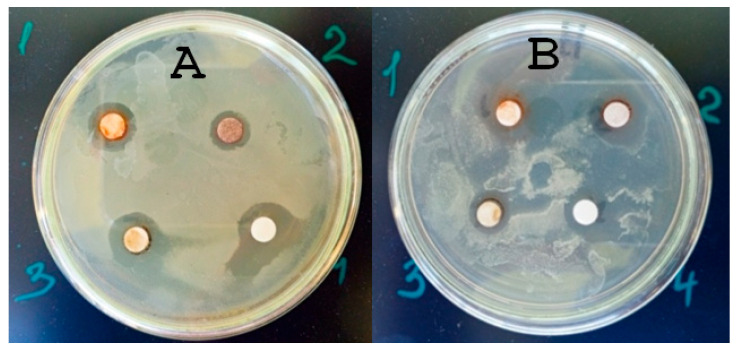
Results of studies of the antimicrobial activity of AgNPs formulations (1—AgNP#1, 2—AgNP#2, 3—AgNP#3, 4—AgNP#4) against bacteria of the following strains: (**A**)—*P. syringae* N1, (**B**)—*P. syringae* N2 by the disk-diffusion method.

The study of the antimicrobial properties of all AgNPs against the phytopathogenic bacteria of two strains of *P. syringae* showed a large degree of antimicrobial activity. Figure 4 presents the results of the disk diffusion test.

As follows from the data in Table 4, the zones of inhibition of phytopathogen growth are from 11 to 13 mm, which is an expression of antimicrobial activity and is consistent with the literature data [44]. It is known that the expression of the toxic, and therefore bactericidal, effect of M-NPs is the formation of reactive oxygen species in living bacterial cells, which damage biomacromolecules, DNA, and cell components, leading to their death [45].

Experimental data on the MBC of AgNPs fully coincided with the data for assessing the MBC of test sanitary indicator microorganisms. The MBC for both strains of *P. syringae* bacteria of AgNP#1–3 was 30 µg/mL, and for the AgNPs#4, 3.5 µg/mL. So, all AgNPs demonstrated antibacterial activity against both *P. syringae* strains, which is consistent with the results for sanitary-indicator microorganisms.

To assess the antimicrobial activity of AgNPs in the soil, a study in the soil contaminated with bacteria of both strains of *P. syringae* was carried out. The soil contaminated with phytopathogens without AgNPs was used as a control. Microbiological control of the total number of *P. syringae* bacteria was made after 3, 7, 10, and 15 days. The results of microbiological analysis are presented in Table 5.

It was noted that the introduction of AgNP#1 into the soil decreased the number of phytopathogens by 5 orders of magnitude in 15 days. A similar effect was observed for AgNP#2. Regarding the formulations AgNP#3 and AgNP#4, the antimicrobial effect was manifested to a higher degree. When these formulations were applied, the presence of phytopathogenic bacteria *P. syringae* was not reliably recorded in the soil after 10 days.

**Figure 5:**
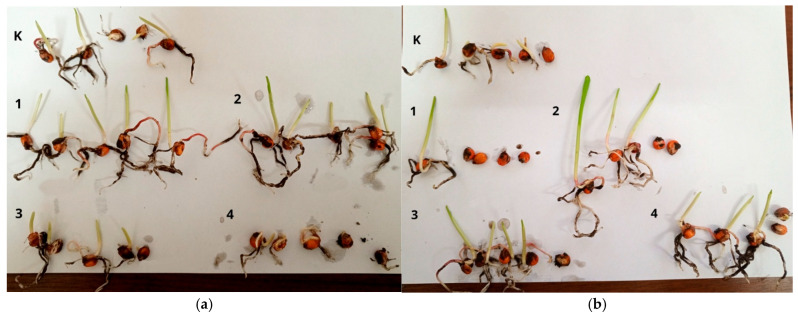
Vegetative tests on germination of *Zea mays* seeds in not contaminated soil (**a**) and soil contaminated with *P. syringae* (**b**) without (K) and with nanosilver formulations: 1—AgNP#1, 2—AgNP#2, 3—AgNP#3, 4—AgNP#4.

The next stage of our research involved studying the nature of *Zea mays* seed germination in the soil contaminated with both strains of *P. syringae* in the presence of the AgNPs formulations. The contaminated soil without nanosilver, as well as the soil not contaminated with *P. syringae,* served as controls. Figure 5 presents the data from the vegetation tests on the germination of corn seeds in soil with and without AgNP#1–4.

In the non-contaminated soil, AgNP#1 and AgNP#2 formulations stimulated the germination of *Zea mays* seeds. The seedlings were larger than in the control, and the number of germinated seeds was the same everywhere (all 5 seeds germinated) (Table 6). Therefore, it can be stated that AgNP#1 and AgNP#2 had a stimulating effect on seed germination, which can be associated with the biological activity of the eucalyptus extract, with the use of which these formulations were obtained. On the other hand, AgNP#4 inhibited the germination of *Zea mays* significantly.

The vegetation tests data on germination of *Zea mays* seeds in the soil contaminated with AgNPs formulations showed a slightly different germination pattern. Although the phytopathogenic strains used in the experiments were isolated from tissues of adult plants, the phytopathogenic effect of *P. syringae* N1 and *P. syringae* N2 was manifested at the stage of seed germination. The seedlings developed extremely slowly and looked morphologically exhausted, as shown by the data in the control groups. It was observed that 4 seeds germinated there. In the *P. syringae*-contaminated soil with AgNP#1, germination of only 1 out of 5 seeds was noted, with AgNP#2, of 3 seeds, with AgNP#3 and AgNP#4, of 4 seeds. The use of AgNP#3 as an antimicrobial agent demonstrated the highest degree of antibacterial action in the soil.

## 4. Discussion

Thus, based on the purpose of this study, namely the evaluation of the antimicrobial potential of biogenically synthesized AgNPs against the sanitary indicator and phytopathogenic bacteria as well as the evaluation of their impact on soil microbiota and plant development, the experimental results showed that all the studied “green” AgNPs exhibited antimicrobial activity against both Gram-positive (GPB) and Gram-negative bacteria (GNB). The working hypothesis assumed that AgNPs obtained by “green” synthesis had sufficient antimicrobial efficacy, while remaining biocompatible with beneficial soil bacteria and non-toxic to plants. The results confirmed this assumption to a large extent and provided new insights into the selective activity of AgNPs under different biological and environmental conditions.

The varying degrees of antimicrobial activity are due to the diverse chemical structure of the synthesized silver NPs. Considering that the syntheses were conducted using different plant extracts and pure tannin, all four types of NPs are different and have different coatings. The coating shell can enhance the antimicrobial effect of some of them. NPs obtained using tannin (AgNP#3) can have a surface coating that enhances the antimicrobial effect. It is known that AgNPs adhere to the surface of bacteria and affect the membrane function [46]. The silver atom is inert and stable, but it becomes reactive when it reaches an oxidation state of Ag^+^. Silver ions (Ag^+^) bind to proteins in the plasma and nuclear membranes, forming a complex that causes structural changes in the membrane [47]. Antimicrobial Ag^+^ is also released through the dissolution of AgNPs. Ag^+^ ions react non-selectively with the electron-donating groups such as thiols, hydroxyls, imidazoles, and phosphates [48]. AgNPs accumulate on the surface of bacteria and form aggregates. As a result, AgNPs create perforations in the cell wall, disrupting the integrity of the membrane, which leads to cell death. It is shown using the example of AgNPs obtained by means of glycosylated polyphenol-flavonoid luteolin, a smaller-sized AgNP is characterized by the strongest antimicrobial activity [49]. The shape and size of nanoparticles can be important even in the case of nanoparticles that have the same area-to-volume ratio. In addition to size and shape, surface modification and heterogeneity affect the antibacterial ability of AgNPs. A recent study showed that surface modification of AgNPs introduces a physical mechanism of antibacterial activity in addition to their inherent ability to interfere biologically [35,50].

In our previous paper [29], a detailed study of the structure of synthesized nanoparticles and their colloidal behaviour was carried out. Table 7 presents the data about the particle size and their ζ-potential.

The synthesized AgNPs displayed diverse size distributions and colloidal properties. The TEM analysis showed core sizes of 5–35 nm, with AgNP#4 exhibiting a bimodal distribution (5 nm and 35 nm), while DLS measurements indicated some degree of aggregation in the aqueous media, particularly for AgNP#1 and AgNP#2 (101–103 nm). The ζ-potentials ranged from −17.5 mV to −35.2 mV, indicating moderate electrostatic stabilization, with AgNP#3 being the most negatively charged (−35.2 mV) and thus most colloidally stable. These physicochemical characteristics correlated with the antimicrobial activity: AgNP#3, which combines a small core size (~17 nm), small aggregation, and large colloidal stability, exhibited the strongest antibacterial effect against *Pseudomonas syringae*. Smaller, stable nanoparticles had a higher surface-area-to-volume ratio and better dispersion, enhancing their interactions with bacterial cells and promoting silver ion release, which likely underlay their superior activity.

The antimicrobial activity observed against both GPB and GNB is consistent with the earlier reports demonstrating the broad-spectrum action of AgNPs [9,12,14]. However, unlike some previous studies that suggested a larger sensitivity of GPB due to the absence of an outer membrane [46,47], our findings did not reveal a significant difference in sensitivity between GPB and GNB. Instead, bactericidal (killing bacteria) and bacteriostatic (inhibiting bacterial growth) effects were observed for the tested AgNPs formulations across all tested strains, regardless of their Gram classification, suggesting that factors such as nanoparticle size, crystallinity, and surface chemistry can play a more defining role than bacterial cell wall structure alone.

Among the tested formulations, AgNP#3 and AgNP#4 demonstrated the most pronounced antimicrobial activity, which was likely due to their smaller particle sizes and larger surface area, facilitating more effective interactions with bacterial cells. The literature supports the importance of nanoparticle size and shape in determining antimicrobial efficiency [36,37,38,39]. Notably, AgNP#4 exhibited the smallest MIC and MBC values, as well as the largest cytotoxicity in the previous biosafety tests [29], which underscored the importance of balancing efficacy with safety for practical applications.

Thus, the antibacterial activity of AgNPs against both GNB (e.g., *E. coli, P. aeruginosa, P. syringae*) and GPB (*S. aureus, E. faecalis*) involves several interrelated mechanisms. Firstly, AgNPs attach electrostatically to cell envelopes, causing structural disruption of the peptidoglycan layer or lipopolysaccharide outer membrane, which increases permeability and leads to leakage of intracellular components [12,19]. Secondly, AgNPs act as reservoirs of silver ions (Ag^+^), which bind thiol groups of essential proteins, inactivating respiratory enzymes and disturbing metabolic pathways [51]. Thirdly, both AgNPs and Ag^+^ induce oxidative stress by stimulating the production of reactive oxygen species (ROS), resulting in lipid peroxidation, protein oxidation, and DNA damage [7]. Moreover, internalized AgNPs and Ag^+^ can interact directly with DNA and ribosomes, inhibiting replication and translation [18]. These mechanisms explain the consistent inhibitory effect of all four AgNP preparations against the tested bacterial strains observed in our MIC/MBC and soil-based experiments.

Notably, the magnitude of antibacterial activity differed among the four green-synthesized AgNP formulations. AgNP#4 (Aloe-based) exhibited the strongest activity (MIC/MBC < 1.5–3.5 µg/mL), which can be attributed to its smallest particle size (<10 nm), enabling rapid release of Ag^+^ and extensive surface interactions with bacterial membranes [34,35]. However, this preparation was also the most cytotoxic, highlighting the trade-off between efficiency and safety. AgNP#3 (tannin-reduced) showed comparably large antibacterial activity with superior colloidal stability, narrow size distribution (10–35 nm), and the highest non-toxic concentration in mammalian cell assays, thus offering the most favourable balance between efficacy and biocompatibility. AgNP#1 (eucalyptus extract, aqueous-ethanolic) demonstrated intermediate effectiveness, consistent with moderate particle size and crystallinity. In contrast, AgNP#2 (aqueous eucalyptus extract) was less active in the agar diffusion assays, likely due to its larger particle size (15–60 nm) and reduced diffusion through solid media. Nevertheless, the liquid culture assays confirmed the antibacterial action of AgNP#2, emphasizing the methodological limitation of diffusion-based assays for nanoparticle evaluation [31,32]. Thus, the most important factor in antibacterial activity is the rate of release of Ag^+^, which depends on the size of AgNPs, their crystal structure, and the phytoshell that reduces and stabilizes the nanoparticles. However, the size of AgNPs also affects their cytotoxicity, which makes their practical application impossible in case of exceeding biosafe concentrations. In the search for an optimal balance between antimicrobial efficacy and biosafety, AgNP#3 (tannin-reduced) showed the best results, whereas AgNP#4 (Aloe-based) were unsuitable for practical use due to their very small particle size and broad size distribution, which make them cytotoxic because of the rapid kinetics of silver ion release and their ability to penetrate living cell membranes.

Besides the size and crystallinity, phytochemical capping agents derived from plant extracts modulate colloidal stability, Ag^+^ release kinetics, and possibly contribute to intrinsic antimicrobial or plant-stimulatory activities [30]. This can explain the observed stimulation of *Zea mays* germination for AgNP#1 and AgNP#2, contrasting with the inhibitory effect of AgNP#4. Finally, the environmental context plays a decisive role: although soil organic matter and ionic strength can limit AgNP dispersion, both AgNP#3 and AgNP#4 were able to eradicate *P. syringae* in soil microcosms completely, whereas AgNP#1 and AgNP#2 reduced bacterial counts by up to five orders of magnitude. Taken together, these results highlight that subtle differences in the synthesis route, particle size, crystallinity, and capping chemistry translate into marked variability in the antibacterial potency and environmental safety profiles of green-synthesized AgNPs.

An important and novel aspect of this study was the assessment of AgNPs’ activity in the soil matrix. AgNP#3 and AgNP#4 suppressed completely *Pseudomonas syringae* populations in the contaminated soil within 10 days, without causing a measurable decline in the total number of beneficial soil microorganisms. On the contrary, both formulations promoted microbial growth, particularly AgNP#3, which increased total bacterial count by 1.74-fold after 15 days. This finding is especially promising in the context of sustainable agriculture, as it suggests that certain “green” AgNPs can sanitize soil from phytopathogens without compromising ecological balance.

Moreover, the vegetative tests with *Zea mays* provided further insight into the biological activity and selectivity of AgNPs. AgNP#1 and AgNP#2, synthesized using eucalyptus extracts, stimulated seed germination in non-contaminated soil. However, only AgNP#3 maintained both antimicrobial efficacy and favourable plant response in the *P. syringae*-infected soil. These results emphasize the potential of AgNP#3 as a candidate for integrated plant protection solutions that combine pathogen suppression with plant growth support.

Taken together, the findings of this study support the hypothesis that green-synthesized AgNPs, particularly AgNP#3, can serve as multifunctional agents for soil sanitation and plant health improvement. These results align with the prior evidence on the role of biologically derived AgNPs in plant protection [14,15,17,20], while also advancing the field by demonstrating their selective action under the actual soil conditions.

Thus, the in vitro studies, in addition to demonstrating the positive properties of AgNPs, such as antimicrobial and antifungal activities, also revealed their adverse and toxic effects on the health of cells or bacteria after exposure to these nanoparticles. The toxicological studies involved exposing a range of cells and organs to different doses of chemicals and monitoring their response over a specified period of time [52].

The antimicrobial properties of silver nanoparticles depend on their size. The small size of silver nanoparticles has an increased active surface area that reacts with bacterial cells, which increases the number of extract molecules attached to the surface [53]. Once released into the environment, AgNPs can undergo various transformations, such as aggregation, agglomeration, or dissolution, resulting in the formation of various chemical compounds (e.g., sulphides or chlorides) [54].

Another transformation of AgNPs in the environment is their combination with various substances, which are predominantly natural organic materials. Depending on the composition of the organic material and the agent forming the nanomaterial, the solubility and aggregation of nanoparticles can vary. For example, the aggregation of AgNPs can be reduced when AgNPs interact with the organic material, forming a coating that stabilises the nanoparticles. Organic compounds found in the environment can also form silver nanoparticles by reducing silver ions [55]. Therefore, nanoparticles in the environment are not found separately, but in combination with various chemical compounds or undergo transformations, which is why their complex toxicity after such transformations should be investigated [56].

Nonetheless, several questions remain open. The mechanisms behind the stimulatory effect of AgNPs on soil microbiota need further clarification, possibly involving microbial community profiling (e.g., via 16S rRNA sequencing). Additionally, long-term studies are needed to evaluate potential cumulative effects, environmental persistence, and the risk of resistance development among soil pathogens. Future research should also explore the formulation of AgNPs into delivery systems such as hydrogels or biochar to enhance their environmental stability and targeted release.

## 5. Conclusions

Thus, in this study, the effective antimicrobial activity of green-synthesized silver nanoparticles (AgNPs) against a broad spectrum of bacteria, including both the sanitary-indicator species (*E. coli*, *S. aureus*, *E. faecalis*, *P. aeruginosa*) and the phytopathogenic strains of *Pseudomonas syringae* isolated from *Zea mays*, was revealed. Among the tested formulations, AgNP#3 and AgNP#4 exhibited the most pronounced bactericidal and bacteriostatic effects, which correlated with their smaller particle sizes and larger specific surface area. A clear relationship was observed between antimicrobial efficiency, particle size, and ζ-potential: smaller and more negatively charged nanoparticles (particularly AgNP#3, −35.2 mV) exhibited stronger antibacterial activity due to better colloidal stability, reduced aggregation, and enhanced interactions with bacterial membranes. Conversely, formulations with larger particles or lower ζ-potential (AgNP#2, −19.2 mV) showed reduced activity, likely because of limited dispersion and slower ion release.

Importantly, the application of AgNPs in the soil environments proved that these nanoparticles could suppress phytopathogenic bacteria effectively without negatively impacting beneficial soil microbiota. Moreover, some formulations, particularly AgNP#3, stimulated overall microbial activity and supported *Zea mays* seed germination in contaminated soil, highlighting their potential for sustainable agricultural use.

These findings underscore the potential of biogenic AgNPs as safe and selective agents for soil sanitation and plant protection. The results also suggest that nanoparticle formulation and synthesis method critically influence not only antimicrobial performance but also biocompatibility and environmental behaviour.

This study emphasizes the significant potential of selected biogenic AgNPs for environmentally safe pathogen control in agroecosystems. Their dual action—antimicrobial and growth-promoting—supports further development toward practical applications in sustainable agriculture and environmental remediation.

## Figures and Tables

**Table 1:** Inhibition zones (mm) of sanitary-indicator bacteria induced by AgNPs.

Test Culture	AgNP#1	AgNP#2	AgNP#3	AgNP#4
*E. coli* ATCC 25922, GNB	11 ± 1	0	10 ± 1	10 ± 1
*E. faecalis* ATCC 29213, GPB	12.5 ± 0.5	0	10	10.5 ± 0.5
*S. aureus* ATCC 25923, GPB	11.5 ± 0.5	8.5 ± 2.5	10.5 ± 0.5	8.5 ± 2.5
*P. aeruginosa* ATCC 27853, GNB	11 ± 1	10.5 ± 0.5	10.5 ± 0.5	11 ± 1

**Table 2:** MIC and MBC values of AgNPs against sanitary-indicator bacteria.

Test Culture	AgNP#1		AgNP#2		AgNP#3		AgNP#4	
	MIC,	MBC,	MIC,	MBC,	MIC,	MBC,	MIC,	MBC,
	µg/mL	µg/mL	µg/mL	µg/mL	µg/mL	µg/mL	µg/mL	µg/mL
*E. coli* ATCC 25922	15	>30	15	23	15	30	1.5	3.5
*E. faecalis* ATCC 29213	15	>30	23	>30	15	30	<1.5	1.5
*S. aureus* ATCC 25923	7.5	30	15	>30	7.5	30	<1.5	1.5
*P. aeruginosa* ATCC 27853	15	>30	15	>30	15	30	1.5	3.5

**Table 3:** Investigations of changes in the number of bacteria in soil with added AgNPs.

AgNP Formulation	Number of Microorganisms, CFU			
	3 Days	7 Days	10 Days	15 Days
AgNP#1	(3.7 ± 0.2) × 10^7^	(3.7 ± 0.1) × 10^7^	(3.5 ± 0.2) × 10^7^	(3.4 ± 0.1) × 10^7^
AgNP#2	(3.5 ± 0.3) × 10^7^	(3.2 ± 0.1) × 10^7^	(3.3 ± 0.2) × 10^7^	(3.3 ± 0.1) × 10^7^
AgNP#3	(4.1 ± 0.1) × 10^7^	(4.8 ± 0.1) × 10^7^	(5.9 ± 0.1) × 10^7^	(6.1 ± 0.1) × 10^7^
AgNP#4	(3.7 ± 0.2) × 10^7^	(4.4 ± 0.1) × 10^7^	(4.9 ± 0.1) × 10^7^	(5.8 ± 0.1) × 10^7^
Control	(3.5 ± 0.3) × 10^7^	(3.4 ± 0.3) × 10^7^	(3.6 ± 0.1) × 10^7^	(3.5 ± 0.1) × 10^7^

**Table 4:** Antimicrobial effect of AgNPs against *P. syringae* bacteria according to the disk-diffusion method and MBC.

AgNP Formulation	*P. syringae* N1		*P. syringae* N2	
	Zone of Inhibition, mm	MBC, µg/mL	Zone of Inhibition, mm	MBC, µg/mL
AgNP#1	13 ± 1	30	12 ± 1	30
AgNP#2	12 ± 1	30	13 ± 1	30
AgNP#3	11 ± 1	30	12 ± 1	30
AgNP#4	12 ± 1	3.5	13 ± 1	3.5

**Table 5:** Changes in the number of phytopathogenic bacteria *P. syringae* (*P. syringae* N1 and *P. syringae* N2) in the soil with added AgNPs.

AgNP Formulation	Inoculation Dose of Bacteria, CFU *	Number of Microorganisms, CFU			
		3 Days	7 Days	10 Days	15 Days
AgNP#1	(1.5 ± 0.1) × 10^7^	(1.2 ± 0.2) × 10^4^	(4.4 ± 0.1) × 10^3^	(1.1 ± 0.1) × 10^2^	(0.8 ± 0.1) × 10^2^
AgNP#2	(1.5 ± 0.1) × 10^7^	(2.2 ± 0.1) × 10^4^	(2.2 ± 0.2) × 10^3^	(1.0 ± 0.1) × 10^2^	(0.5 ± 0.1) × 10^2^
AgNP#3	(1.5 ± 0.1) × 10^7^	(2.3 ± 0.2) × 10^4^	(4.3 ± 0.1) × 10^3^	0	0
AgNP#4	(1.5 ± 0.1) × 10^7^	(1.6 ± 0.1) × 10^4^	(1.3 ± 0.1) × 10^3^	0	0
Control **	(1.5 ± 0.1) × 10^7^	(1.1 ± 0.1) × 10^7^	(1.7 ± 0.1) × 10^5^	(1.1 ± 0.1) × 10^5^	(1.0 ± 0.1) × 10^4^

* CFU—Colony forming unit; ** Control—the soil not treated with AgNPs, but kept under the conditions similar to those used in the experiment–time, temperature.

**Table 6:** Study of *Zea mays* seed germination in clean and phytopathogen-contaminated soils containing AgNPs.

AgNP Sample	Uncontaminated Soil		Soil Contaminated with *P. syringae*	
	Number of Germinated Seeds, Units	Length of the Seedling, mm	Number of Germinated Seeds, Units	Length of the Seedling, mm
AgNP#1	5	36 ± 12	1	64 ± 18
AgNP#2	5	34 ± 11	3	34 ± 29
AgNP#3	5	31 ± 5	4	36 ± 7
AgNP#4	5	6 ± 2	4	31 ± 8
Control	5	17 ± 4	4	11 ± 4

**Table 7:** Average particle size (D_ef_) and their ζ-potential AgNPs [29].

AgNP Sample	D_ef_, nm		ζ-Potential, mV
	According to TEM *	According to DLS **	
AgNP#1	16 nm	101 nm	−28.3
AgNP#2	28 nm	103 nm	−19.2
AgNP#3	17 nm	7 nm, 30 nm	−35.2
AgNP#4	5 nm, 35 nm	10 nm, 93 nm	−17.5

* TEM—Transmission Electron Microscopy. ** DLS—The dynamic light scattering method.

## Data Availability

The original contributions presented in this study are included in the article. Further inquiries can be directed to the corresponding authors.

## Abbreviations

The following abbreviations are used in this manuscript:

AgNPs	Silver Nanoparticles
ATCC	American Type Culture Collection (bacterial strain repository)
CFU	Colony-Forming Unit (used in microbiology to quantify viable bacteria)
DNA	Deoxyribonucleic Acid
GNB	Gram-Negative Bacteria
GPB	Gram-Positive Bacteria
LA	Luria Agar (nutrient medium for bacterial culture)
MBC	Minimum Bactericidal Concentration (lowest concentration of an antimicrobial agent that kills bacteria)
MIC	Minimum Inhibitory Concentration (lowest concentration of an antimicrobial agent that inhibits bacterial growth)
M-NPs	Metallic Nanoparticles
PCR	Polymerase Chain Reaction (a technique for amplifying DNA)
Psy	Pseudomonas syringae-specific phytopathogenic gene
ROS	Reactive Oxygen Species (highly reactive molecules causing oxidative stress)
NPs	Nanoparticles
TEM	Transmission Electron Microscopy (a technique for imaging at the nanoscale)
*Zea mays*	Scientific name for maize (corn)
